# Phenethylisothiocyanate Potentiates Platinum Therapy by Reversing Cisplatin Resistance in Cervical Cancer

**DOI:** 10.3389/fphar.2022.803114

**Published:** 2022-04-25

**Authors:** Elizabeth Mahapatra, Debomita Sengupta, Ravindra Kumar, Budheswar Dehury, Salini Das, Madhumita Roy, Sutapa Mukherjee

**Affiliations:** ^1^ Department of Environmental Carcinogenesis and Toxicology, Chittaranjan National Cancer Institute, Kolkata, India; ^2^ School of Biotechnology, National Institute of Technology Calicut, Kozhikode, India; ^3^ ICMR-Regional Medical Research Centre, Chandrasekharpur, Bhubaneswar, India

**Keywords:** PEITC, chemosensitization, cisplatin resistance, MRP2, PI3K/AKT

## Abstract

Acquired cisplatin resistance in cervical cancer therapy is principally caused by reduction in intracellular drug accumulation, which is exerted by hyperactivation of the oncogenic PI3K/Akt signaling axis and overexpression of cisplatin-exporter MRP2 along with prosurvival effectors NF-κB and IAPs in cervical cancer cells. These activated prosurvival signaling cascades drive drug efflux and evasion of apoptosis for rendering drug-resistant phenotypes. Our study challenges the PI3K/Akt axis in a cisplatin-resistant cervical cancer scenario with phenethylisothiocyanate (PEITC) for chemosensitization of SiHa^R^, a cisplatin-resistant sub-line of SiHa and 3-methylcholanthrene–induced cervical cancer mice models. SiHa^R^ exhibited higher MRP2, p-Akt^Thr308^, NF-κB, XIAP, and survivin expressions which cumulatively compromised cisplatin retention capacity and accumulated PEITC better than SiHa. SiHa^R^ appeared to favor PEITC uptake as its accumulation rates were found to be positively correlated with MRP2 expressions. PEITC treatment in SiHa^R^ for 3 h prior to cisplatin exposure revived intracellular platinum levels, reduced free GSH levels, generated greater ROS, and altered mitochondrial membrane potential compared to SiHa. Western blot and immunofluorescence results indicated that PEITC successfully downregulated MRP2 in addition to suppressing p-Akt^Thr308^, XIAP, survivin, and NF-κB expressions. In mice models, administration of 5 mg/kg body-weight PEITC priming dosage prior to treatment with 3 mg/kg body-weight of cisplatin remediated cervical histology and induced tumor regression in contrast to the group receiving the same dosage of cisplatin only. This suggested PEITC as a potential chemosensitizing agent in light of acquired cisplatin resistance in cervical cancer and established its candidature for Phase I clinical trial.

## Introduction

Cervical cancer, the fourth-leading cause of morbidity among women worldwide (Globocan, 2018), is reported to cause maximum deaths among Indian women (NICPR, 2018). Relapse and recurrence catered by failure in treatment owing to acquirement of resistance to chemo/radiotherapy is a common occurrence (Dasari and Tchounwou, 2014; D’Alterio et al., 2020). Conventionally, chemotherapy with platinum-based drugs such as cisplatin, alongside other chemotherapeutics or radiation (Concurrent Chemoradiotherapy; CCRT), is used for treating invasive cervical cancer (Stage IIB onward), where cisplatin is used as a radiosensitizer (Todo and Watari, 2016). Therefore, loss of cisplatin sensitivity with eventuation of resistance can be highly detrimental (Zhu et al., 2016).

Cisplatin (cis-diamminedichloroplatinum; CDDP) renders its action by attacking DNA to generate complex irreparable DNA adducts following ‘intracellular hydrolytic activation’ (Lorusso et al., 2014). Pleomorphically, cancer cells evade drug effects through some epigenetic and genetic changes that get triggered upon drug treatment (Kim et al., 2018). Similar changes also desensitize cervical cancer cells to CDDP for augmenting resistance (Seol et al., 2014). Overcoming CDDP resistance for improved treatment is the utmost requisite for better therapy outcome.

The Phosphatidylinositol 3 Kinase/Protein Kinase B or Akt (PI3K/Akt) signaling axis plays a pivotal role in conferring cells with CDDP-resistant properties by metabolically transforming them for reducing drug accumulation (Muggia et al., 2015). AKT/protein kinase B, being serine threonine kinases, gets activated by PI3K upon induction by various extracellular triggers such as chemotherapeutic drugs (Shi et al., 2019). Activated phospho-Akt1 inhibits IκB to evoke NF-κB for promoting evasion of apoptosis by triggering Inhibitor of Apoptosis proteins (IAPs) (Fu et al., 2014; Barra et al., 2019). This prosurvival axis domineers the event of acquired CDDP resistance by translationally activating CDDP exporters, namely, P-glycoprotein-1(Pgp1) and Multidrug Resistance Protein (MRP2), which conveniently pump out CDDP and critically reduce its intracellular levels, thereby restraining apoptosis (Zhang et al., 2020; Liu et al., 2021). Targeting of MRP2 and its upstream regulators, that is, PI3K/Akt axis, therefore, may provide a therapy-rationale for cervical cancer.

Natural phytochemicals from cruciferous vegetables such as phenethylisothiocyanate (PEITC) potentiate apoptosis by negatively regulating prosurvival molecules (Wang et al., 2011; Gupta et al., 2014; Dai et al., 2016). Reports suggest that PEITC can induce apoptosis in cancer cells to enable resistance reversal (Sarkar et al., 2012; Biswas et al., 2021) ***.*** PEITC chemosensitizes many cancer cells by scavenging free radicals (Soundararajan and Kim, 2018). In biliary tract cancer cells, PEITC was reported to induce apoptosis *via* glutathionylation-dependent degradation of Mcl-1 (Li et al., 2016). Additionally, in gastric cancer cells, PEITC was reported to inhibit Multidrug Resistance gene (MDR1), MRP1, Akt, and NF-κB (Tang et al., 2014). Thus, mechanistic insight into PEITC-mediated targeting of PI3K/Akt signaling may be considered for improvising the therapy and prognosis of cervical cancer. The present study aims to explore the mechanism of PEITC-mediated regulation of this prosurvival signaling axis for overcoming acquired CDDP resistance in cervical cancer scenarios.

## Materials and Methods

### Cell Culture

SiHa cells were maintained in Minimum Essential Medium (MEM) supplemented with 10% fetal bovine serum (FBS) and antibiotics (gentamycin 40 μg, penicillin 100 units, and streptomycin 10 μg/ml) at 37°C in a humidified CO_2_( ∼ 5%) incubator.

A CDDP-resistant subline (SiHa^R^) was developed from the parental SiHa cell line by ‘pulse treatment’ (Sharma et al., 2010). Thereafter, the doubling time was calculated. Dissimilarities in cellular morphology were studied under a phase-contrast microscope (Olympus). Respective protocols have been elaborated in Supplementary Data S1.

### Animal Experimentation

A cervical cancer mice model, developed by chronic 3methylcholantherene (3MC; chemical carcinogen) treatment of virgin female Swiss Albino mice (*Mus musculus*; 5–6 weeks old; weight: 23–25 gms), was used in this study (Mahapatra et al., 2020). All animals were obtained from the Central Animal Facility of CNCI and housed in polyvinyl cages within well-ventilated rooms (temperature: ∼ 22°C; relative humidity: 50–60%; 12 h day/night cycle). The details of animal acclimation have been provided in Supplementary Data S1.

Based on body weight, 50 mice were randomized into five broad groups (Group IV), each consisting of ten animals separated into two batches [No. of mice (n) = 5/cage x 2]. Group I was kept as an “untreated” control group where mice did not receive any treatment. Invasive cancer-bearing mice were randomized into Group II (no intervention), Group III (3 mg/kg body weight CDDP), Group IV (2.5 mg/kg body weight PEITC), and Group V (PEITC followed by CDDP). These doses were selected after proper dosimetry. During the concurrent chemocycles of two weeks, body-weight fluctuations and tumor-growth alterations were recorded periodically. Food and water were provided *ad libitum*.

### MTT Assay

The MTT assay was performed in SiHa and SiHa^R^ cells after exposure to a wide range of PEITC (SIGMA Aldrich) and CDDP (CIPLA) concentrations as per the detailed description of the process provided in Supplementary Data S1.

### Cyclocondensation Assay

Enumeration of the ‘optimum period’ and quantitation of intracellular PEITC in SiHa and SiHa^R^ following 1, 2, 3, 4, 5, and 6 h of its administration was undertaken by the cyclocondensation assay (Zhang and Talalay, 1998). SiHa and SiHa^R^ cells were treated with 2 µM of PEITC (maximum tolerated dose or MTD). PEITC concentrations were determined by spectrophotometry against a standard curve (Supplementary Data S3). The experimental discourse has been described in Supplementary Data S1.

### Flameless Atomic Absorption Spectroscopy

The frozen cell pellets (-20°C) of SiHa and SiHa^R^ were brought to room temperature, lysed in radio-immunoprecipitation assay lysis buffer (RIPA), and acid-digested in concentrated nitric acid at 60°C (Federici et al., 2014) for 2 h. All samples were quantitated for platinum levels at an absorbance of 265.9 nm in an inert argon gas chamber supplied with a platinum lamp being operated at 10 mA current. The measurements were recorded against varied concentrations (0.1 nM-25 µM) of platinum using an atomic absorption spectrometer (VARIAN). Each experiment was repeated five times.

### Assessment of Cell Viability by Trypan Blue Dye Exclusion Assay

Calculation of cell viability in SiHa and SiHa^R^ was performed following the trypan blue dye exclusion method. Accordingly, the cells were pelleted down by centrifugation at 1,500 rpm for 5 min after trypsinization. Equal volumes of cell suspension and 0.4% trypan blue stain were thereafter incubated for 1 min, followed by differential counting of the live and dead cells under a Phase Contrast Microscope (Olympus) using a hemocytometer. The results were graphically (% viable cells *vs*. treatment points) represented. The experiments were repeated thrice.

### Histopathological Study

The dissected mice cervix tissues were washed in cold normal saline (0.87%), fixed in 10% neutral buffered formalin (NBF; MERCK), and processed for histology sectioning (Mahapatra et al., 2020).

### Flow Cytometry and Fluorescence Microscopy

Intracellular ROS was quantitatively estimated by flow cytometry (BD FACS Calibur; BD Biosciences) followed by qualitative analysis under a fluorescent microscope. Equal densities of SiHa and SiHa^R^ were seeded in 55-mm plates (2.5 × 10^6^ cells) and over coverslips in 6-well plates (2.5 × 10^5^ cells) for flow cytometry and microscopic analysis, respectively. The plates were trypsinized and incubated with 10 μM 2′,7′-dichlorofluorescein dihydroacetate (DCFH-DA; Santa Cruz) for 45 min, followed by flow cytometric analysis in FL1-H. Scatter plots and histograms were generated in replicates using Cell Quest software. Respective coverslips from the corresponding 6-well plates were scanned for qualitative analysis of generated ROS using an FITC filter under a fluorescent microscope (Leica).

### Rhodamine 123 Assay

SiHa and SiHa^R^ cells, seeded in densities of 2.5 × 10^5^ cells/well in 6-well plates, were stained with 5 μg/ml of rhodamine (Rh-123) and incubated at 37°C for 30 min following PEITC and CDDP treatment. The results were generated spectrofluorimetrically (VARIAN; excitation-488 nm, emission-525 nm) and represented graphically (fold-change) for each replicate point.

### Estimation of Free GSH Level

Free glutathione reductase [E.C1.8.1.7] levels were spectrophotometrically assessed as per the protocol of the glutathione assay kit (Cayman Chemical) for triplicate experimental sets.

### Western Blotting

The expression status of prosurvival effectors (Akt/p-Akt, NF-κB, XIAP, and survivin) and cisplatin exporter pump (MRP2) was comparatively studied by Western blotting after standardized laboratory protocol (Mahapatra et al., 2020). The particulars of the antibodies and inhibitors used have been provided in Supplementary Data S1.

### Semi-Quantitative Reverse Transcription PCR Analysis

Isolation of total cellular RNA was performed using TRIzol reagent (Invitrogen). cDNA was synthesized from 2 μg of total RNA using a RetroScript kit (Ambion/Applied Biosystem) which was amplified by PCR using respective forward and reverse primer sequences (Supplementary Data S1). The PCR product was analyzed by electrophoresis in ethidium bromide (EtBr) containing 2% agarose gel and visualized under a gel documentation system.

### Immunofluorescence

The cells were seeded (2.5 × 10^5^ cells/well) onto coverslips placed within 6-well plates for performing immunocytochemistry. PEITC and CDDP-treated cells were immunostained with respective antibodies and fluorophore-tagged secondary antibodies as per laboratory protocol (Biswas et al., 2021).

### Cytopathological Study

The smears of cervical exfoliated cells suspended in PBS were fixed with 100% ethyl alcohol and stained as per the protocol given by Mahapatra et al. (2020), followed by microscopic (ZEISS) analysis.

### Systemic ROS Quantitation

Reactive oxygen species (ROS) generated due to chronic treatment with 3 MC in animal models were spectrofluorimetrically quantitated according to the protocol given by Biswas et al. (2010), which is detailed in Supplementary Data S1.

### *In Silico* Studies

AutoDock Vina (Trott and Olson, 2010) was utilized in all the docking experiments with the optimized protein models as the docking target against the ligand PEITC. The detailed protocol is enclosed in Supplementary Data S1.

### Statistical Analysis

The mean values of the PEITC, CDDP, and PEITC + CDDP points were compared by factorial analysis of variance (ANOVA). The relationship between the studied parameters was analyzed by calculating Pearson’s correlation coefficient using the CORREL function of Microsoft Excel. Data were expressed as mean ± standard deviation (S.D.) A *p*-value < 0.0001 was considered statistically significant.

## Results

### Elevated Expression of MRP2 in SiHa^R^ Facilitates Better Intracellular Uptake and Accumulation of PEITC

**FIGURE 1:**
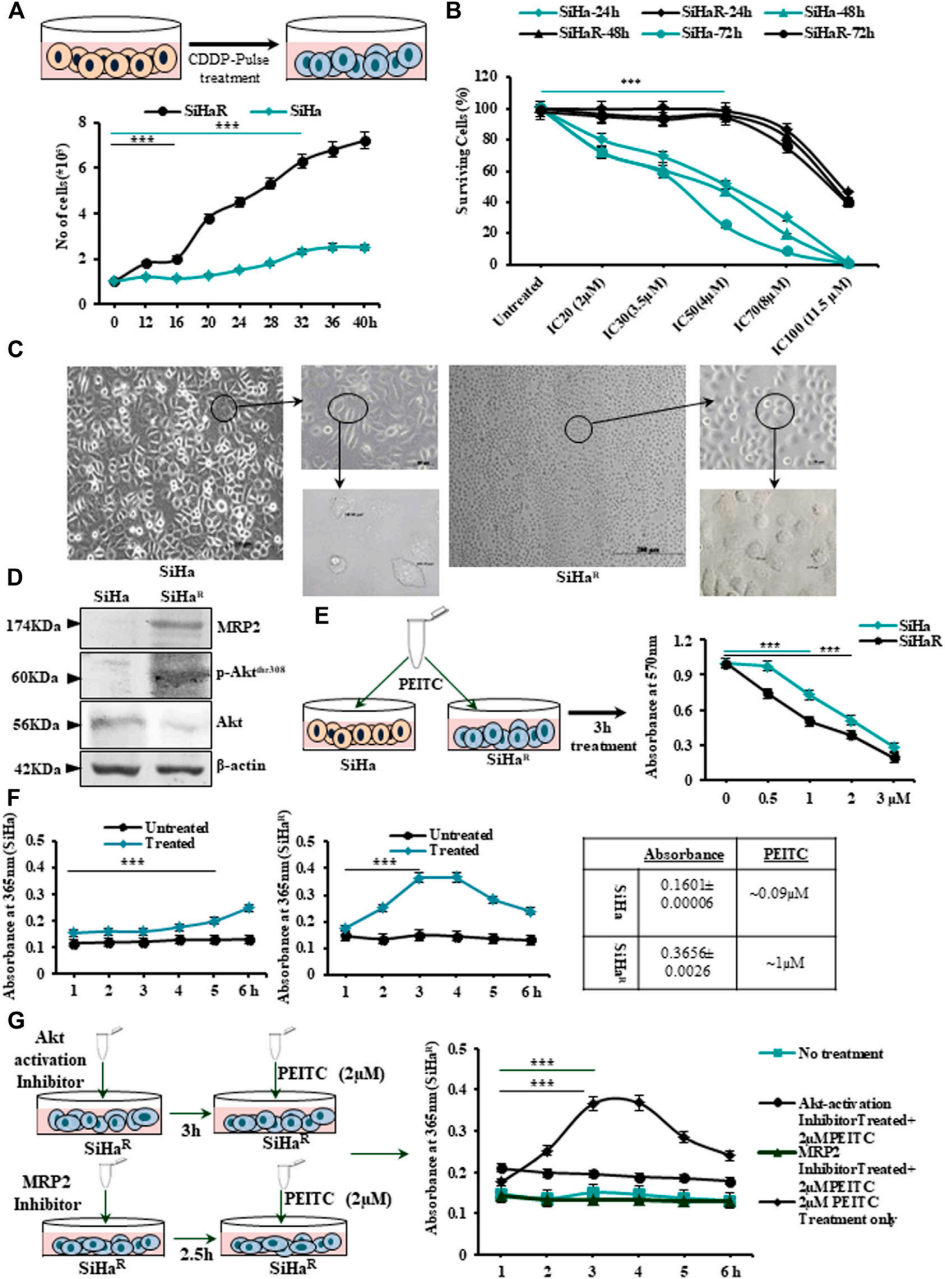
**(A)** Schematic outline of the “pulse treatment” methodology followed for the development of cisplatin (CDDP)-resistant subline SiHa^R^ from the parental CDDP-sensitive SiHa cell line (upper panel). Representation of the cell-growth patterns depicting differences in the doubling time (SiHa-32h and SiHa^R^-16 h) in a graphical format (lower panel). **(B)** Graphical anecdote of the MTT survivability assay results for SiHa and SiHa^R^ cells following treatment with CDDP for 24, 48, and 72 h with IC_20_ (2 µM), IC_30_ (3.5 µM), IC_50_ (4 µM), IC_70_ (8 µM), and IC_100_ (11.5 µM) CDDP doses of SiHa at 24 h. **(C)** Phase-contrast micrographic snippets of SiHa and SiHa^R^ representing remarkable differences in their morphology. Magnification for the main image is ×100 (Scale bar: 100 µm), while that of the subsequent insets are 200x (Scale bar: 50 µm) and ×400 (Scale bar: 20 µm). **(D)**Western blot results depicting differential expression patterns of Akt, p-Akt^Thr308^, and MRP2 proteins in SiHa and SiHa^R^. Respective band intensities as calculated by ImageJ software have been provided in Supplementary Data S2. **(E)** Pictorial demonstration of the experiment and the subsequent graphical representation of MTT assay results for identifying the respective IC_50_ doses of phenethylisothiocyanate (PEITC) in SiHa and SiHa^R^ cells. **(F)** Graphical description of the findings of cyclocondensation assay performed to quantitate intracellular PEITC uptake levels in SiHa and SiHa^R^. **(G)** Pictorial emulation (left panel) with corresponding graphical representations (right panel) of cyclocondensation assay performed particularly with SiHa^R^ after treatment with Akt and MRP2 inhibitors, respectively. All experiments **(A–G)** were repeated thrice. The values have been represented as the mean of three independent determinants (Mean ± SD), where ^***^p represented *p* < 0.0001 compared to untreated cells.

The finally isolated subline SiHa^R^, developed by “pulse treatment” of SiHa, displayed terminal resistance to the parental IC_30_ (3.5 µM) dose of CDDP (Figure 1A upper panel). SiHa^R^ exhibited proliferative enhancement, as evident from the graph (Figure 1A, lower panel). The resistant subline SiHa^R^ doubled within 16 h, unlike SiHa, which doubled in 32 h (Figure 1A, lower panel). Consequently, the surviving potential of SiHa^R^ was validated by the MTT assay, wherein SiHa^R^ cells showed appreciable survival in the parental IC_20_ (2 µM), IC_30_ (3.5 µM), and IC_50_ (4 µM) CDDP concentrations for 24, 48, or even 72 h, respectively. Death of SiHa^R^ cells in comparison to SiHa at IC_70_ (8 µM) and IC_100_ (11.5 µM) CDDP doses was notably less (Figure 1B). Relatively, the calculated IC_70_ and IC_100_ doses of CDDP in SiHa turned out to be the IC_30_ and IC_60_ in case of SiHa^R^, while the IC_50_ of CDDP for SiHa^R^ was calculated to be around 11 µM. Therefore, SiHa^R^ was estimated to be 2.75-fold resistant to CDDP.

Furthermore, a comparative morphological characterization of SiHa and SiHa^R^ by microscopic examination (Figure 1C) vividly delineated morphological differences among the sublines. SiHa^R^ had an increased nucleus-to-cytoplasmic ratio (666.81 µm^2^) compared with that of SiHa (367.97 µm^2^). As per Western blot results (Figure 1D), SiHa^R^ expressed remarkably higher levels of MRP2 (2.1 fold) and p-Akt^Thr308^ (1.91 fold), unlike the parental SiHa. A significantly higher ratio of p-Akt/Akt (1.7 fold) in SiHa^R^ cells **(** Supplementary Data S2A,B **)** indicated the contributory role of the upregulated PI3K/Akt pathway in the acquirement of the CDDP-resistant phenotype.

Before exploring the mechanistic role of PEITC in overcoming acquired CDDP resistance, it was important to calculate the growth inhibitory dosage of PEITC in SiHa and SiHa^R^ cells. The MTT assay was therefore undertaken following treatment of cells with PEITC doses ranging between 0.5 and 3 µM for 3–12 h (data not shown). Accordingly, the time-point of 3 h was selected (Figure 1E) in which the IC_50_ dose of PEITC was calculated to be 1 and 2 µM for SiHa^R^ and SiHa, respectively. This indicated that 50% killing in SiHa^R^ got mediated by exactly half of the PEITC IC_50_ dose of SiHa cells. This was supported by spectrophotometric results of the cyclocondensation assay, where SiHa^R^ was found to accumulate higher intracellular PEITC (0.3656 ± 0.0026; 1 µM) than SiHa (0.1601 ± 0.00006; 0.09 µM) for 3 and 4 h. (Figure 1F). Treatment extension for 5 h yielded reduced absorbance of 0.2836 ± 0.00021 (0.7 µM) in SiHa^R^ cells (Figure 1F). However, a subtle rise (0.2 ± 0.0005; 0.5 µM) in PEITC level among SiHa cells was noted after 5 h (Figure 1F). Apparently, SiHa^R^ accumulated PEITC better than SiHa.

When these experiments were repeated with SiHa^R^ following treatment with MRP2 and Akt inhibitors, interesting observations were attained (Figure 1G). Inhibition of Akt activation compromised PEITC accumulation by SiHa^R^, unlike its usual nature. Surprisingly, upon MRP2 inhibition, the uptake reduced significantly (Figure 1G). These findings affirmed that increased PEITC uptake in SiHa^R^ was a result of higher MRP2 expression.

### PEITC Expedited CDDP Retention and Enabled CDDP-Mediated Intracellular ROS Generation to Curb the Growth of Resistant Cervical Cancer Cells

**FIGURE 2:**
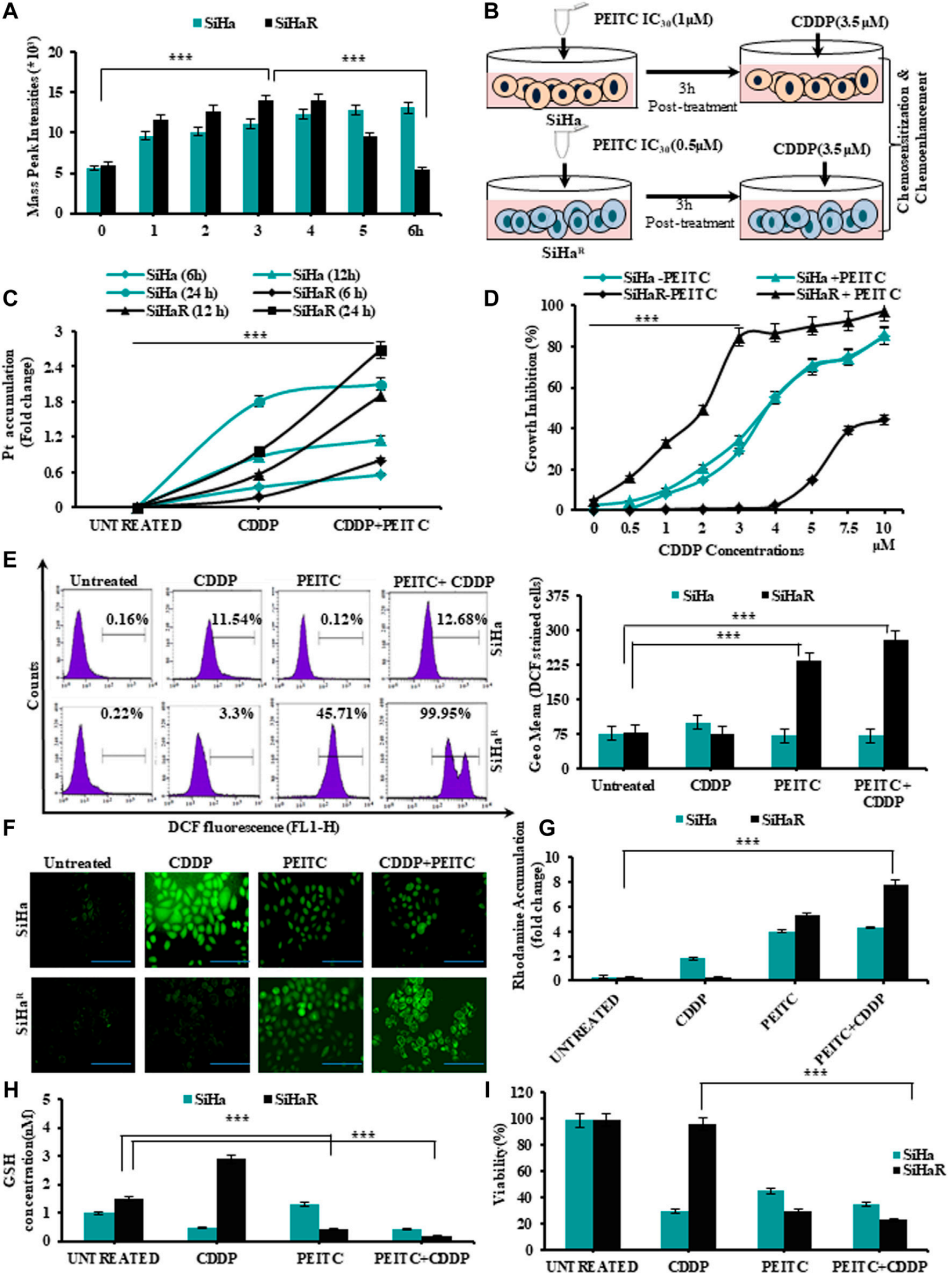
**(A)** Mass spectroscopic analysis to identify the optimum time point of PEITC uptake and retention in CDDP-sensitive and -resistant cells. **(B)** Schematic outline of the entire treatment design followed for validating the effects of PEITC as a cisplatin chemosensitizer and chemoenhancer. **(C)**Time-dependent intracellular accumulation of CDDP in SiHa and SiHa^R^ cells upon exposure to 3.5 µM of the drug following PEITC (IC_30_ dose) pre-treatment for 3 h. **(D)** Comparative growth inhibition of SiHa and SiHa^R^ in a varied range of CDDP doses for 24 h in absence and presence of PEITC pretreatment for ascertaining optimum combinatorial dose. **(E)** Histogram plots displaying FL1 peak shifts due to ROS generation represented by DCFHDA stained cells (SiHa^R^ and SiHa) subjected to treatment with PEITC and CDDP either in a pretreatment mode or solely (left panel). The geometric mean (Geo.Mean) values of these DCFHDA stained cells were plotted graphically (right panel). **(F)** Relative fluorescent microscopic images showing ROS content of SiHa^R^ and SiHa cells treated either with PEITC or CDDP or both (Magnification: ×400; Scale bar: 20 µm). **(G)** Graphical representations of the spectrofluorimetric findings corresponding to mitochondrial membrane potential status of SiHa^R^ and SiHa with reference to rhodamine 123 dye accumulation. **(H)** Kit-based spectrophotometric quantification of free GSH levels in SiHa^R^ and SiHa. Absorbance values were recorded for five kinetic cycles at an interval of 1 min at 340 nm. **(I)** Relative trends of cellular viability (%) as determined by the trypan blue dye exclusion method. All the experiments **(A–I)** were performed in triplicate, and the values were expressed as mean ± SD; ^***^ *p* < 0.0001 with respect to untreated cells.

In alignment with the spectrophotometric findings of Figure 1F **,** mass spectroscopic analysis of PEITC-treated SiHa and SiHa^R^ for the same time intervals was performed in order to identify the retention time of PEITC (Figure 2A). Respective mass-peak intensities of the cyclocondensed intracellular PEITC intermediate (1, 3-benzodithiol 2 thione) were found to be significant for SiHa^R^ in 3 h, which sustained till 4 h, followed by a decline. PEITC levels in SiHa could only be detected from 3 h and onward. Conclusively, the results permitted the selection of the 3 h time-point as the ‘optimum treatment time’ in successive experiments.

Therefore, SiHa and SiHa^R^ were treated with 3.5 µM of CDDP following a 3 h pretreatment with their respective IC_30_ PEITC doses (SiHa: 1µM; SiHa^R^: 0.5 µM) for exploring the association between their PEITC accumulation and CDDP-retention capacities (Figure 2B). Intracellular platinum levels, as quantified by flameless atomic absorption spectroscopy (Figure 2C), revealed an improved and increasing trend in the drug retention capacities of PEITC-enriched SiHa^R^ cells with respect to PEITC-deficient SiHa for 24 h. In addition, PEITC pretreatment could also efficiently restrain SiHa^R^ growth in even higher CDDP doses, wherein it would normally grow in the absence of the phytochemical (Figure 2D). As evident from the graphical anecdote (Figure 2D), 50% of PEITC pretreated SiHa^R^ got killed by only 2 µM of CDDP, while the same for SiHa cells was achievable with a higher CDDP concentration of about 3.7 µM. These results highlighted the chemosensitizing potentials of PEITC.

Mechanistic insights of the chemoenhancing potentials of PEITC were further explored by checking the cell-killing ability of the retained CDDP by generating ROS *via* disruption of mitochondrial membrane potential followed by depletion of free-GSH levels. Depictions of flow cytometry results (Figure 2E, left panel) portrayed a clear peak shift for DCF generation in CDDP-treated SiHa^R^ cells in the presence of PEITC pretreatment as compared to SiHa. The frequency of DCF-bearing SiHa^R^ cells was greater than that of SiHa for combinatorial treatment modalities (Figure 2E, right panels). In addition, the findings were strengthened by the corroborating fluorescent microscopic results representing the ROS content of SiHa and SiHa^R^ cells (Figure 2F). In fact, the relative mitochondrial-membrane potential was found to be highly disrupted in the rhodamine 123 staining assay (Figure 2G) because SiHa^R^ cells accumulated rhodamine 16.7 times more than SiHa in a combination treatment setup. Interestingly, the free-GSH levels of SiHa^R^ were reduced by 0.39 folds upon CDDP treatment only in the case of prior PEITC priming, unlike SiHa, which showed no noteworthy alterations (Figure 2H). Finally, the trypan blue dye exclusion methodology revealed a significant ROS-mediated reduction in SiHa^R^ viability to 66% (*p* < 0.0001) from 98.5% upon PEITC treatment ahead of CDDP treatment (Figure 2I). All these results also entitled PEITC as a CDDP chemoenhancer in SiHa^R^ cells.

### PEITC Increased the Efficacy of CDDP by Negatively Regulating Prosurvival Markers and Drug Exporter MRP2

**FIGURE 3:**
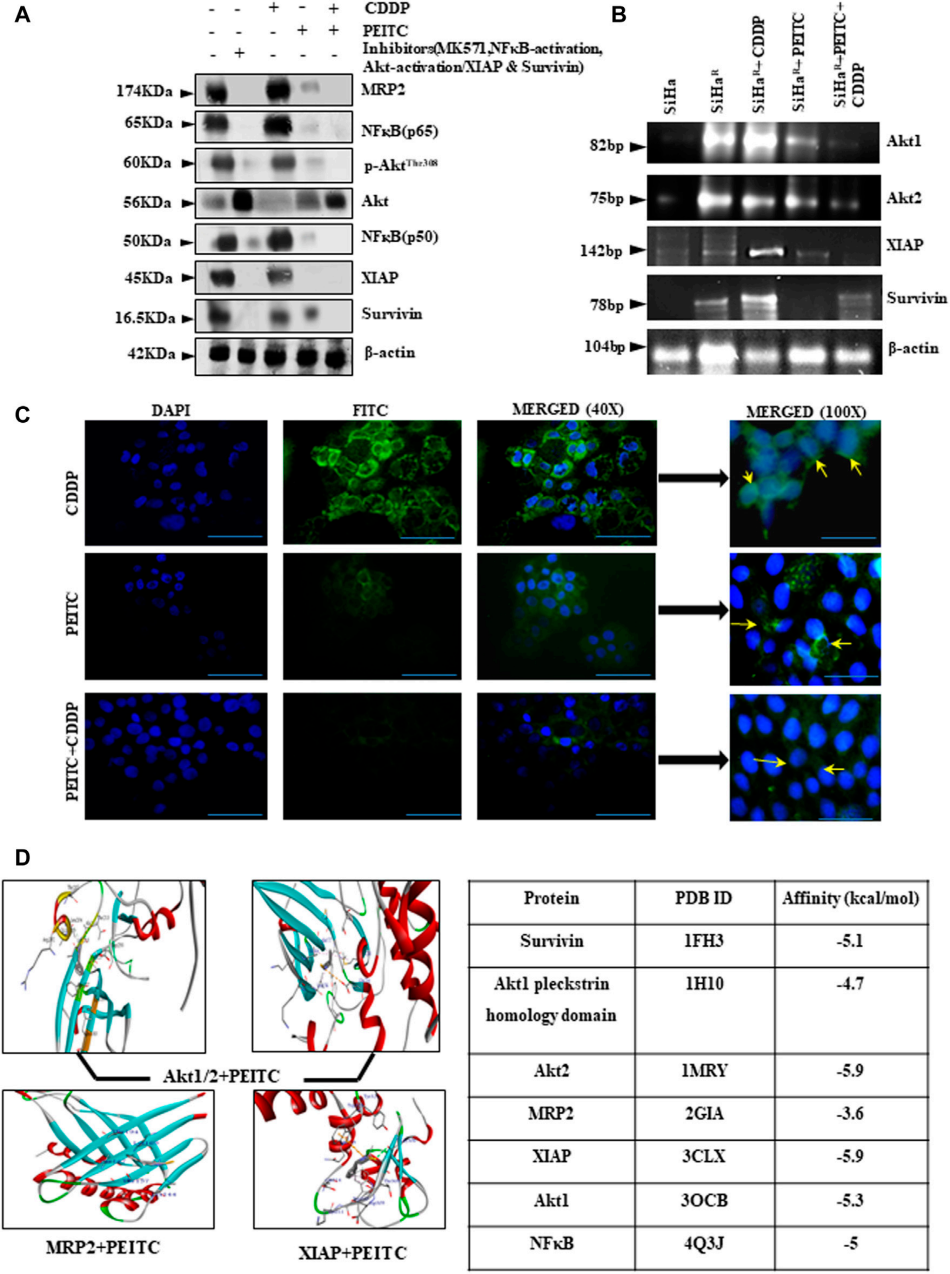
**(A)** Relative protein expressions of Akt/p-Akt^Thr308^, NF-κB (p50/p65), MRP2, XIAP, and survivin by Western blotting. β-actin was used as a loading control. Respective band intensities of these proteins have been represented graphically in Supplementary Data S4. **(B)** Relative effects of PEITC (pre-treatment) along with CDDP upon Akt and IAP mRNAs as represented in the RT-PCR blots. Lane # 1: SiHa, Lane # 2: SiHa^R^, Lane# 3: SiHa^R^ + CDDP (3.5 µM), Lane # 4: SiHa^R^ + PEITC (0.5 µM), Lane # 5: SiHa^R^ + PEITC (0.5 µM) + CDDP (3.5 µM) **(C)** Spatiotemporal distribution of MRP2 upon similar treatment conditions in SiHa^R^ and SiHa cells as displayed by indirect immunofluorescence data. Main images are magnified up to ×400, while the insets displayed alongside are magnified up to 1,000x. Scale bar of each field measures to about 50 µm (main images) and 20 µm (side-insets). About 50 fields were scanned under the microscope for data acquisition. **(D)** AutoDock Vina results highlighting PEITC interaction with Akt, MRP2, and survivin in the best poses. Scores of all the possible interactions have been incorporated in tabular format. Images displaying PEITC interactions with other prosurvival molecules have been provided in Supplementary Data S5. All experiments were performed in triplicate.

In an effort to investigate the regulatory effect of PEITC over deregulated proteins (Akt, NF-κB, MRP2, XIAP, and survivin) for chemoenhancing CDDP, the SiHa^R^ cells were treated with the respective pharmacological inhibitors alongside differential treatment with either CDDP or PEITC or both. The proteins isolated thereafter were comparatively studied for deciphering the inhibitory role of PEITC. Western blot results (Figure 3A) depicted remarkable decrease in the expression patterns of pAkt^(Thr308)^, total-Akt, XIAP, survivin, NF-κB (p65), NF-κB (p50), and MRP2 in combinatorial treatment modalities of PEITC and CDDP in comparison to single-agent treatments and untreated SiHa^R^. Respective band intensities (Supplementary Data S4) also revealed that the results were comparable with pharmacological inhibition of the respective molecular markers (Figure 3A). This inhibition was not delimited at protein levels of these cells as the RT-PCR blots (Figure 3B) suggested a depleted expression of the relative mRNAs. Moreover, PEITC was observed to directly affect the subcellular localization of MRP2 in SiHa^R^. As portrayed in immunofluorescence micrographs (Figure 3C), PEITC restricted MRP2 localization in the nuclear periphery of SiHa^R^ cells which was originally found in their membrane in the presence of sole CDDP treatment. On the contrary, neither the cell membranes nor the nuclear periphery harbor any MRP2 in SiHa^R^ when subjected to CDDP after PEITC pretreatment (Figure 3C).

PEITC was screened for the top-ranked poses based upon the docking score and non-bonded contact potential with the target protein conformations. Docking results (Figure 3D) delineated the highest affinity between PEITC and Akt2 (-5.9 kcal/mol) and XIAP (-5.9 kcal/mol). Specifically, the Thr213 residue of Akt2 formed a hydrogen bond, while its Leu204 formed pi–pi electrostatic interaction with PEITC. XIAP exhibited one hydrogen bond (Gly306), three pi–pi electrostatic, and two electrostatic interactions with Gln319 and Trp323 residues. An overall strong affinity was also observed between NF-κB and PEITC (-5 kcal/mol). Detailed portrayal of considerable interactions between PEITC and other molecules also supported the notion (Supplementary Data S5).

### PEITC Successfully Restricted Tumor Growth in an *In Vivo* Model by Augmenting CDDP Responses

**FIGURE 4:**
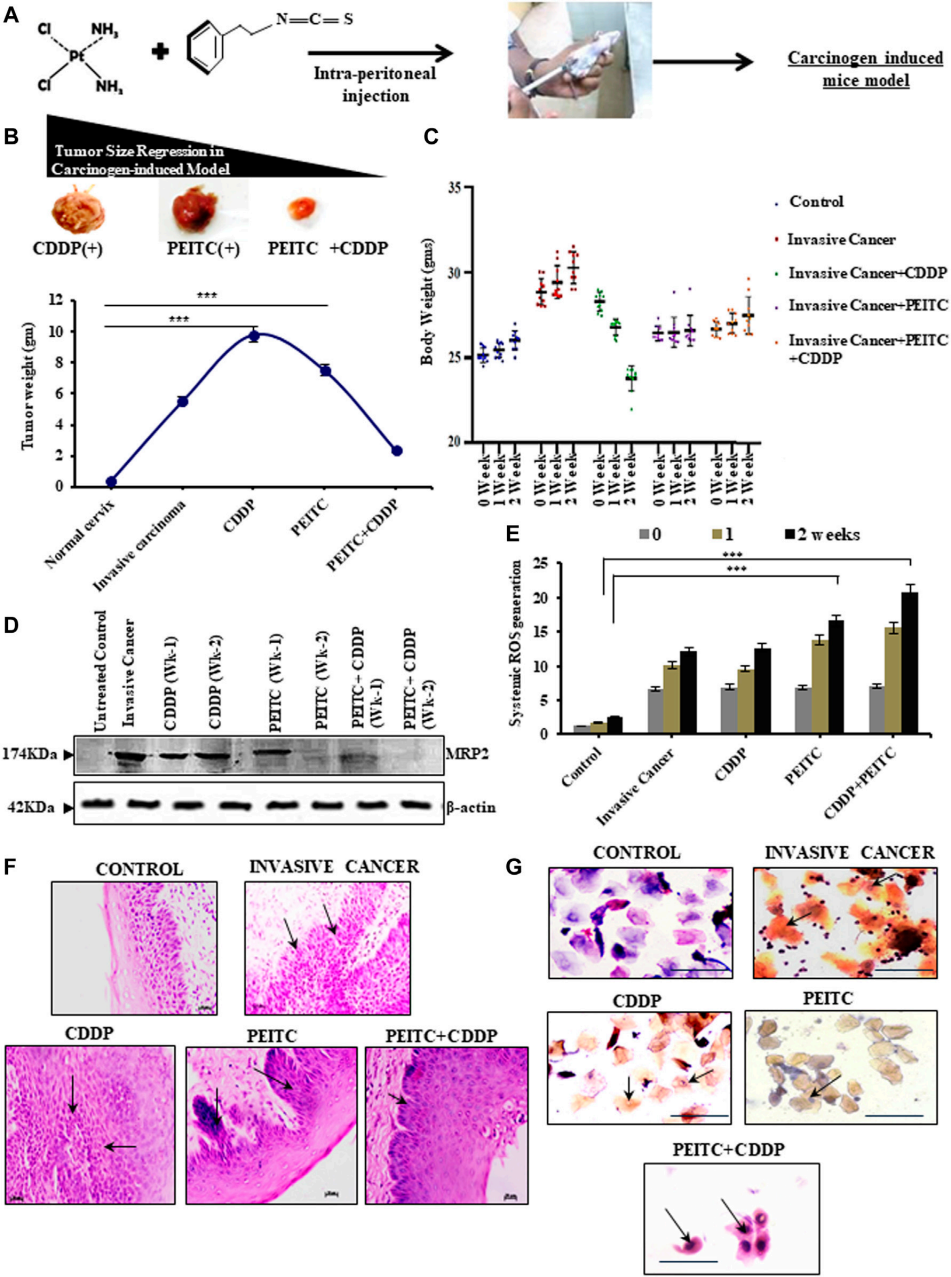
**(A)** Clear outline of the treatment protocol observed for animal experimentations in a carcinogen-induced cervical cancer model. **(B)** Pictorial (upper panel) and corroborative graphical descriptions (lower panel) of tumor size regression among invasive cancer-bearing mice after treatment with CDDP or PEITC or CDDP + PEITC. **(C)** Trends in body-weight alterations in mice upon interventions with PEITC (5 mg/kg body weight) and CDDP (3 mg/kg body weight) either in combination or solely with respect to untreated healthy control and non-intervened invasive cancer-bearing mice. **(D)** Western blot results depicting the modulatory effect of PEITC upon the expression profiles of MRP2 proteins in the carcinogen-induced cervical tumor microenvironment in the isolated or combination mode. **(E)** Graphical representation of the systemic ROS levels upon PEITC and CDDP. **(F)** Histopathological (left panel; Magnification: ×400; Scale bar: 20 µm) and **(G)** cytopathological changes (right panel; Magnification: ×400; Scale bar: 20 µm) represented by hematoxylin–eosin and PAP staining, respectively, displaying the effect of the treatment regimen. Marked areas (arrow indicated) in histology demonstrate alterations in epithelial growth within the stroma, while cellular keratinization and nuclear–cytoplasmic ratio variations within cervical exfoliated cells in cytology have been delineated (arrow indicated). Numerical results were displayed as mean ± SD; ^***^ *p* < 0.0001 with respect to untreated. All experiments **(B–G)** were performed in triplicate.

The chemoenhancing effect of PEITC in an *in vivo* invasive cervical cancer model was explored by intraperitoneal administration of CDDP and PEITC either in combination or singularly **(** Figure 4A). Pictorial and graphical representations showed that combination treatment efficiently regressed tumor size (Figure 4B **,** upper panel) and subsequently decreased its weight (Figure 4B **,** lower panel) among the treatment groups. Nevertheless, sole treatment with 3 mg/kg body weight of CDDP failed to reduce the tumor size as the tumor weight remained unaltered, reflecting the acquirement of potential drug-insensitive nature. The results corroborated with the harmonizing patterns in their relative body weight fluctuations (Figure 4C).

These findings prompted an investigation of the protein-expression status of MRP2. For this purpose, the tumor cortex, stroma, and cortico-medullary regions were differentially studied for the expression profiles of MRP2, both with and without PEITC interventions during CDDP chemocycles of two weeks. Interestingly, MRP2 levels were characteristically high in all the tumor regions (Figure 4D). However, 2.5 mg/kg body weight of PEITC decreased these increasing expression patterns of MRP2, which went unchecked in the presence of sole CDDP intervention (Figure 4D). This observation verified the *in vitro* findings which claimed that effective PEITC function was an outcome of MRP2 upregulation. Elevations in systemic ROS levels were noted in these combinatorial treatment groups, indicating direct control of PEITC administration over CDDP-mediated free-radical generation (Figure 4E). Histopathology (Figure 4F) and cytopathology (Figure 4G) of these groups supported the findings as administration of PEITC in individual and in combination with CDDP was found to restrict the invasive basal cells from intruding the stromal region of the cervical epithelium, besides checking their extensive keratinization (Figure 4G).

## Discussion

Resolving the problem of acquired CDDP resistance by finding specific chemosensitizers is the utmost need of the hour. In this regard, natural isothiocyanates can be considered better options due to the exploitation of drug exporters for cellular entry. Paramount reports suggested that efflux pumps (Pgp, MRP1/2 and BCRP) promote the intracellular import of isothiocyanates (Telang et al., 2009; Mi et al., 2011; Morris and Dave, 2014). PEITC, in this study, was found to chemosensitize MRP2 overexpressing CDDP-resistant SiHa^R^ cells, which also promoted its intracellular uptake and retention. Hence, PEITC in this study was identified as a better cell growth inhibitor for SiHa^R^ than SiHa. Previous experimental evidences have firmly established PEITC as an anticancer, chemopreventive (Chen et al., 2012; Ioannides and Konsue, 2015; Suvarna et al., 2017), and chemosensitizing agent (Yang et al., 2014; Souvick et al., 2020). Correlatively, SiHa^R^ cells, which grew prolifically in 3.5 µM of CDDP owing to acquired CDDP resistance, had surprisingly ceased to grow in the same and higher drug doses following PEITC pretreatment for 3 h. This permitted negation of relative toxicity of CDDP, which often impedes therapy. This pre-treatment improved drug-retention capacities of SiHa^R^ and thereafter delivered adequate growth inhibition. However, PEITC-primed SiHa cells exhibited no enhancement in their platinum levels, and their viability remained unaffected. This corroborated with the preferential PEITC accumulation in SiHa^R^ over SiHa cells. Chemosensitization is just not enough for the reversal of acquired chemoresistance among aggressive cancers as their deregulated molecular signaling conveniently deteriorates the chemotherapeutic pharmacodynamicity. ROS overproduction in SiHa^R^ cells upon PEITC treatment (sole/combinatorial) supported the abovementioned viewpoints and aligned with pre-existing reports (Hong et al., 2015). The present study recorded high free-GSH levels in CDDP-treated SiHa^R^ cells which went down manifold when 3 h of PEITC pre-treatment was ensued. This abided with reports suggesting the prevalence of inverse correlation between increased cellular GSH levels and CDDP-accumulation (Okazaki et al., 2019; Liu et al., 2021) as the drug also attacks GSH for allowing cellular cytotoxic death by free-radical outburst (Achkar et al., 2018). Among SiHa cells, tracer amounts of PEITC could not mediate pro-oxidant functions neither in the presence nor in the absence of CDDP. This necessitated the importance of “exposure-time” and “exposure-dose” of PEITC in relaying antioxidant or pro-oxidant functions in a cell-specific manner. Mitochondrial membrane potential disruption furthermore confirmed that PEITC acted as a CDDP chemoenhancer in SiHa^R^ cells.

Considering the pioneering role of the PI3K/Akt signaling cascade in orchestrating the scenario of acquired CDDP resistance in cervical cancer, it was intended to concentrate upon the ways in which PEITC modulated this cascade for altering MRP2 distribution in SiHa^R^ particularly. It was observed that the phosphorylation status of Akt had remarkably reduced in SiHa^R^ with PEITC followed by CDDP treatment. Expression profiles (protein/mRNA) of downstream effectors of the signaling cascades aligned with those of p-Akt. The expression profiles of NF-κB (p50/p65) decreased, resulting in cumulative inhibition of XIAP, survivin, and MRP2. PI3K/Akt is reported to ubiquitously modulate the multidrug-resistant phenotype in cancer (Zhang et al., 2020; Liu et al., 2021). Therefore, shutting down the activity of the upstream effectors would concoct the acquired resistant phenotype of cancer cells. Accordingly, the spatiotemporal distribution of MRP2 proteins was apparently disturbed. PEITC pretreatment for 3 h among SiHa^R^ cells considerably reduced MRP2 accumulation in the membrane even upon CDDP treatment. This unveiled the root cause of increasing CDDP levels within the resistant cells upon PEITC pretreatment. A milieu of reference studies also reported that apparently, PEITC reduces the expression of drug exporters to promote the reversal of acquired chemoresistance (Morris and Dave, 2014; Suvarna et al., 2017). *In silico* observations pinpointing at specific interactions of PEITC with Akt, XIAP, and MRP2 proteins further established the role of PEITC as a chemosensitizer.

Upon validation in a 3 MC induced *in vivo* cervical cancer model, reiterations of similar results were attained. The mice group when treated with CDDP alone did not restrict the tumor growth, as evident from histopathological study and tumor images. However, prior PEITC administration alongside CDDP injection controlled tumor growth and improved the relative histology and cytology by permissibly surging systemic-ROS levels. These aggregated evidences were enough to confirm PEITC as a CDDP sensitizer in cisplatin-resistant cervical cancer. Although, detailed insight into PEITC-mediated cisplatin sensitization in *in vivo* set-up is mandatory. The present study has laid down a foundation for the candidature of PEITC as a cisplatin sensitizer and enhancer in Phase I clinical trial.

## Data Availability

The original contributions presented in the study are included in the article/Supplementary Material, further inquiries can be directed to the corresponding author.
