# Supplementary material for: Phenethylisothiocyanate Potentiates Platinum Therapy by Reversing Cisplatin Resistance in Cervical Cancer
Source: Front Pharmacol. 2022 Apr 25;13:803114. doi: 10.3389/fphar.2022.803114 (PMC9081374; doi:10.3389/fphar.2022.803114)
Supplement: Supplementary file 1 [file DataSheet1.PDF]

**Supplementary Data 1**

**Development of CDDP resistant subline**

As a pre-requisite, SiHa cells were treated with a wide range of CDDP doses (0.1-200 μM) and MTT assay was performed to identify the $IC_{20}$ (2 μM), $IC_{30}$ (3.5 μM), $IC_{50}$ (4 μM) and $IC_{70}$ (8 μM) doses of the drug for the cell line. In order to develop a CDDP resistant subline from parental SiHa, 'pulse treatment' procedure was observed. Initially, parental SiHa cells were allowed to transform from a transient chemotolerant phenotype to a permanent chemoresistant phenotype through exposure to three consecutive pulses of specific CDDP concentrations followed by drug-free cell maintenance for a 14days period of 'drug holiday' after which the surviving cells were again challenged with the same drug concentration for verification of its acquired-resistant phenotype. Accordingly, dose escalation was undertaken with sub-culturing being performed for 5-6passages for every dose-pulse. Each pulse of CDDP was provided once a week (single-shot drug treatment once a week, i.e., three shots in three weeks) and thereafter a CDDP resistant subline of SiHa named $SiHa^R$ was developed over a period of 18months. A very low CDDP-dose of 0.1 μM ($1/40^{th}$ of $IC_{50}$) was employed primarily for pulse treatment initiation while final isolation of sub-line was made at 3.5 μM or the $IC_{30}$ dose. Fold Resistance (F.R) was calculated as the ratio of $IC_{50}$ of resistant cells to $IC_{50}$ of parental cells [F.R=( $IC_{50}$ of $SiHa^R$)/( $IC_{50}$ of SiHa)] for determining the relevance of resistance acquired by the subline.

**Calculation of doubling-time of SiHa and $SiHa^R$**

SiHa and $SiHa^R$ cells were seeded in densities of $1*10^5$ cells within 6 well plates and were allowed to grow for the time periods of 12,16,20,24,28,32,36 and 40h respectively. At respective time points, cells from the allocated wells were trypsinized and counted using haemocytometer under inverted microscope (Nikon) for enumeration of the cell number. For every time instant, cell number was calculated in a triplicate setup. Results were represented as Mean ±S.D.

**Animal Maintenance**

Prior to treatment initiation, the animals were subjected to a two-week acclimation period during which all the female and male mice were kept in complete isolation from each other. This induced pheromone influenced oestrous cycle synchrony thereby nullifying hormonal interference (Mc Clintock et al., 1981).

**MTT Assay**

In order to identify CDDP doses prior to 'pulse treatment' initiation, SiHa cells of density 10,000/well were seeded in 96-well plates for each points in triplicate fashion followed by maintenance at $37^0$C for 24h, 48h and 72h respectively. Except for control, every other set were treated with CDDP doses ranging between 0.1 μM to 200 μM for different time-points and eventually incubated with MTT (6mg/5 ml) reagent (SIGMA Aldrich) for 4 h. In due course, 170μl of MTT containing supernatant was discarded after centrifugation at 1000rpm for 10 min at $4^0$C followed by DMSO addition with mild shaking for another 10 min. The blue-coloured formazan product formed was detected at 570 nm in an ELISA plate reader

(TECAN). Thereafter, the survival rates of $SiHa^R$ in the parental $IC_{20}$, $IC_{30}$, $IC_{50}$ and $IC_{70}$ doses were accordingly studied for 24, 48 and 72h respectively.

Similar protocol was also followed for separately identifying the PEITC doses for treating SiHa and $SiHa^R$ sublines after subjecting them to varied 0.5, 1, 2 and 3 μM doses of the phytochemical for 3h. Additionally, absorbances were also recorded at 570 nm for identifying the optimum growth inhibitory concentrations of cisplatin in combination with PEITC.

**Cyclocondensation Assay**

This methodology involved quantification of intracellular PEITC levels in terms of 1, 3Benzodithiole-2-thione which is the PEITC-intermediate formed during cyclocondensation reaction. Generated intermediate is quantified spectrophotometrically (VARIAN) at 365nm. The accumulation levels of the specific concentrations of the intermediate with a molecular weight of 184.30g were also additionally verified by mass spectroscopy in terms of the detectable mass-peaks within the cell-lysates harvested at specific time points. Spectrophotometric absorbances were plotted against a standard curve for calculating PEITC levels in μM/ μg of protein. Intracellular presence of respective concentrations of PEITC was validated by Liquid Chromatography with tandem mass spectrometry (LC-MS-MS; WATERS). Each experiment was undertaken in triplicate and results were expressed as Mean ±S.D.

**Western blot analysis**

Some of vital reagents/antibodies used for western blot analysis along with specific inhibitors used are as follows:

Akt activation inhibitior (SIGMA Aldrich), NFκB activation inhibitior (Merck Millipore), XIAP inhibitor-Dequalinium chloride (SIGMA Aldrich), MRP2 inhibitor-MK571(SIGMA Aldrich) and Survivin inhibitor (Merck Millipore) were used for validation of the inhibitory role of PEITC. The primary antibodies used were Akt (GeneTex, 1:1000), $p\text{-}Akt^{(Thr308)}$ (abcam, 1:1000), NFκB (p50/p65) (GeneTex, 1:1000), XIAP (GeneTex, 1:1000), survivin (GeneTex, 1:1000), MRP2 (abcam , 1:1000), and β-actin (GeneTex, 1:1000).

**Semi-quantitative reverse transcription PCR analysis (RT-PCR)**

The primer sequences used for generating RT-PCR results are as follows:

**1) Akt 1:**
Forward primer CAGGAGGTTTTTGGGCTTG
Reverse primer TGAGGAAGACAGGACCAGGA
**2) Akt 2:**
Forward primer AAAGAAGGCTGGCTCCACAA
Reverse primer GTCGCTCTTCAGCAGGAAGT
**3) XIAP:**
Forward primer ACTTCGGGTTTCACGACTCC
Reverse primer CCGAGCCCCAATCTGGAAAT

**4) Survivin:**
Forward primer CCCTCACTGCTGAAGGACAC
Reverse primer GACAGCCCTCACTCCCTAGA

**5) β-actin:**
Forward primer GACAGTCAGCCGCATCTTCT
Reverse primer GCGCCCAATACGACCAAATC

**Estimation of systemic ROS levels in isolated blood leukocytes of mice**

One volume of mouse blood collected aseptically from the heart was mixed with three volumes of Solution A (pH-7.2; 0.87% $NH_4Cl$ in 10 mM Tris HCl), incubated on ice for 20 min and centrifuged at 400 g for 20 min at 0°C. The supernatant was discarded, and the pellets were again resuspended in Solution A followed by centrifugation at 400 g for another 20 min at 0°C. The resulting pellets were suspended in Solution B (pH-7.2; 0.25 M mesoinositol, 10 mM $Na_2SO_4$, 1 mM $MgCl_2$), cold centrifuged at 1500 rpm for 5 min at $4^0$C, and resuspended in HEPES-buffered saline (HBS; pH 7.4; 140 mM NaCl, 5 mM KCl, 10 mM HEPES, 1 mM $CaCl_2$, 1 mM $MgCl_2$, 10 mM glucose). Cell numbers were adjusted to $10^6$ cells/ml/point in HBS which were incubated with a fluorescent probe, 2',7'-dichlorofluorescein dihydroacetate or DCFH-DA (10 μM, Sigma-Aldrich, USA), for 45 min at room temperature in complete darkness. DCFH-DA passively diffuses within cells to transform into a diol moiety, which is further oxidized into a fluorescent compound, 2', 7'-dichlorofluorescein (DCF), by intracellular ROS. DCF was quantitated spectrofluorimetrically (VARIAN; Excitation: 485 nm and Emission: 530 nm). For each point, readings were recorded approximately five times in triplicate attempts of the experiment. Results were expressed as Mean ±S.D.

***In silico* studies**

Molecular docking was applied to see the various interactions of PEITC (PubChem ID: 16741) with all the molecules AKT (PDB ID: 1H10), AKT1 (3OCB), AKT2 (1MRY), MRP2 (2GIA), NFκB-p65 (4Q3J), XIAP (3CLX) and Survivin (1F3H). Each protein structure was prepared by the removal of water molecules and co-crystalized ligands and subsequent addition of polar hydrogen atoms. Similarly the compound PEITC was also prepared by addition of 7.5 hydrogen atoms using Avogadro tool. AutoDock Vina software was utilized in all the docking experiments, with the optimized protein models as the docking target. For each target protein, individual runs were performed. Ranking of the resultant docking poses were screened using a built-in scoring functions. By utilizing the AutoDock tools, different size grid box were employed with center of the grid box being placed around the active site of each protein for docking using AutoDock Vina. The screened poses were analyzed using BIOVIA Discovery Studio Visualizer and PyMOL.

A

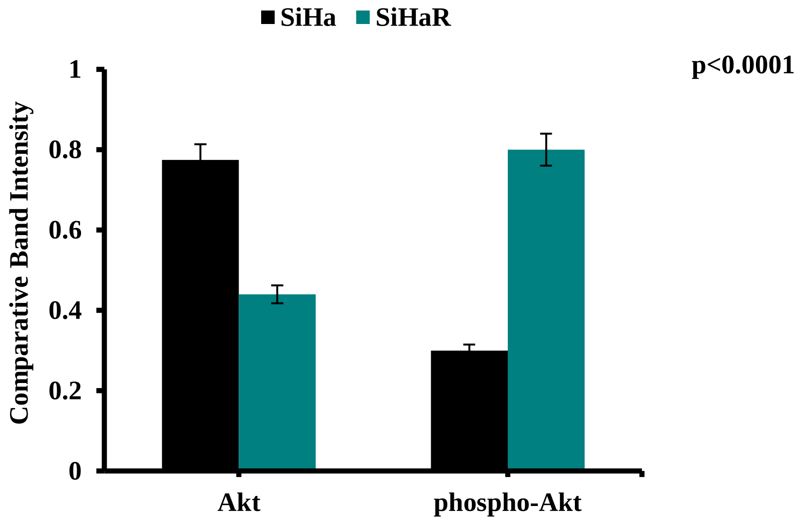


B

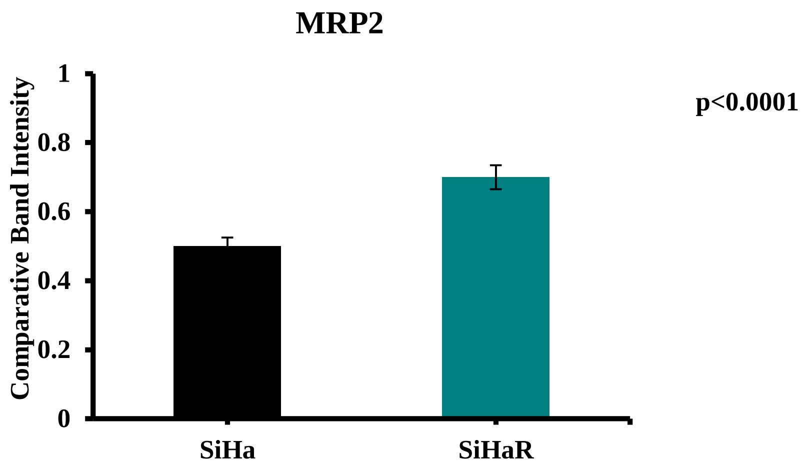


**Supplementary Data 2: (A & B) Comparative band intensity patterns of Akt, p-Akt and MRP2 in SiHa and SiHa$^{R}$ after normalization against the loading control β-actin**

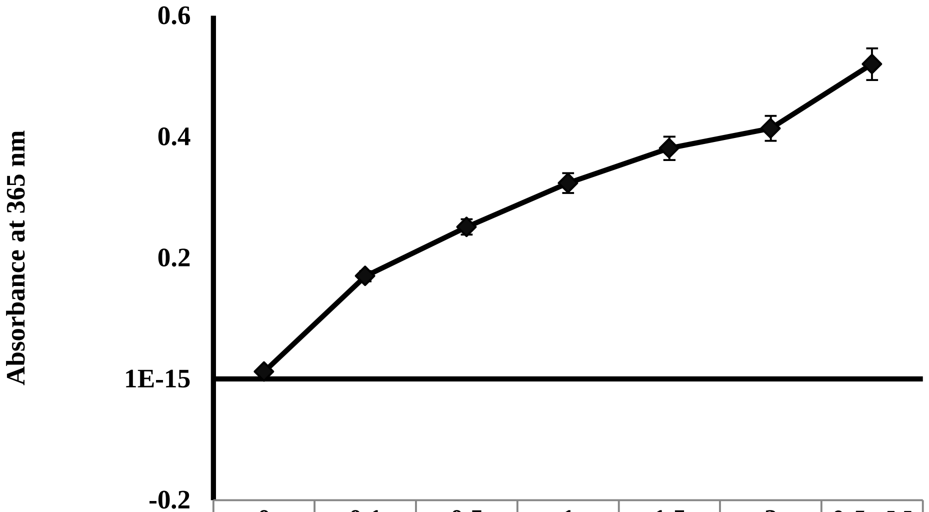


| | 0 | 0.1 | 0.5 | 1 | 1.5 | 2 | 2.5 μM |
|---|---|---|---|---|---|---|---|
| Series1 | 0.012 | 0.17 | 0.2511 | 0.3236 | 0.381 | 0.414 | 0.52 |

**1, 3Benzodithiole-2-thione concentration (μM)**

**Supplementary Data 3: Standard Curve of the PEITC intermediate 1, 3Benzodithiole-2-thione.**

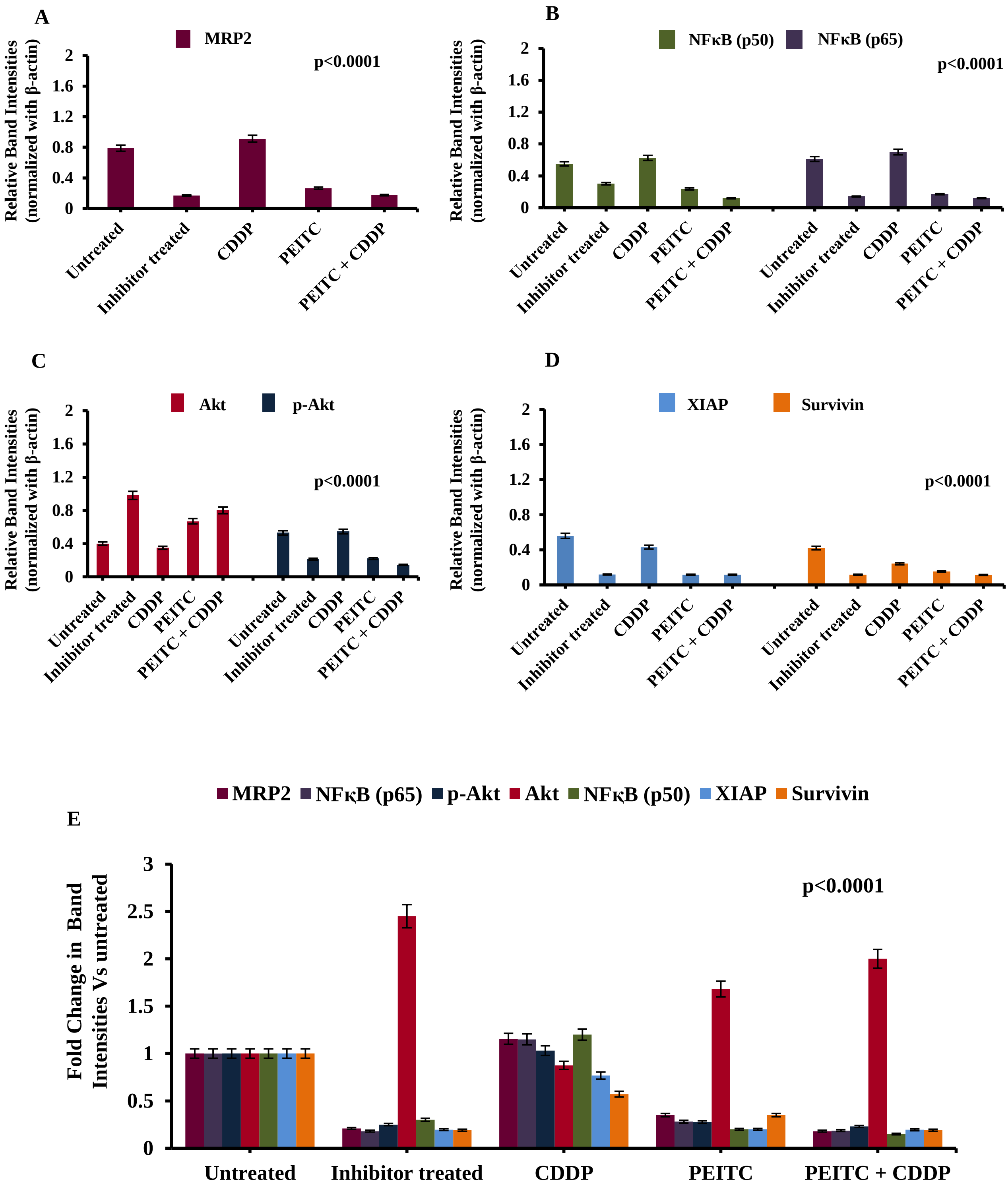


**Supplementary Data 4: (A-D) Relative band intensities of the prosurvival markers (Akt, p-Akt, NFκB, XIAP, Survivin and MRP2) expressed graphically after normalization against β-actin. (E) Comparative anecdote of the expressions patterns of these prosurvival markers with respect to untreated SiHa$^{R}$.**

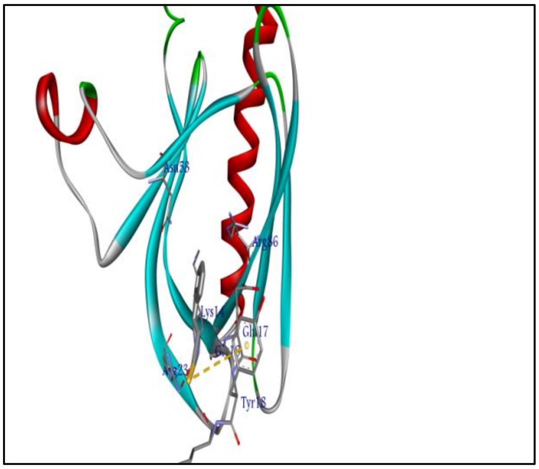

**Akt (PH) domain + PEITC**

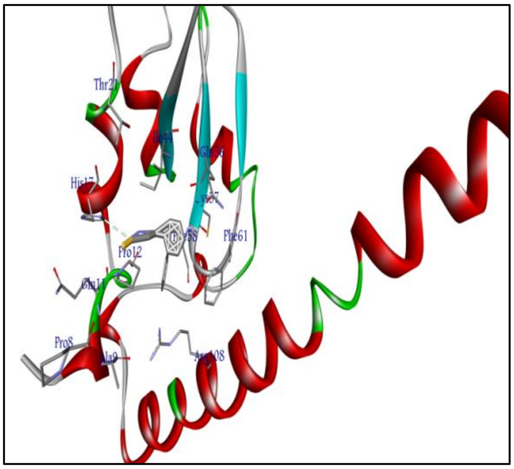

**Survivin + PEITC**

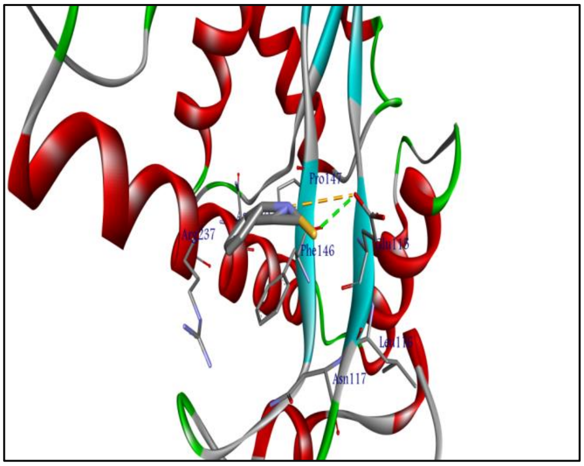

**NFκB+ PEITC**

**Supplementary Data 5: PEITC interaction with the best poses of Akt (PH) domain , Survivin and NFκB.**
